# Modulation of inflammatory factors predicts the outcome following spinal cord injury

**DOI:** 10.1186/s13018-020-01727-1

**Published:** 2020-06-01

**Authors:** Zepeng Yu, Xingwei Sun, Rui Xia, Qian Chen, Qin Wu, Weiwei Zheng

**Affiliations:** 1 Department of Intervention, The Second Affiliated Hospital of Soochow University, Suzhou, 215004 People’s Republic of China; 2 Department of Oncology, The Second Affiliated Hospital of Soochow University, Suzhou, 215004 People’s Republic of China; 3 Department of Oncology, Affiliated Suzhou Hospital of Nanjing Medical University, Suzhou, 215008 People’s Republic of China; 4 Department of Ultrasonography, Suzhou Science and Technology Town Hospital, Affiliated Suzhou Hospital of Nanjing Medical University, Suzhou, 215001 People’s Republic of China; 5 Department of Orthopaedics, Affiliated Suzhou Hospital of Nanjing Medical University, Suzhou, 215008 People’s Republic of China

**Keywords:** Spinal cord injury, Inflammatory responses, Basso-Beattie-Bresnahan scores, IL-4, IL-10

## Abstract

**Background:**

The correlation between inflammatory responses caused by spinal cord injury (SCI) and the prognosis of patients with SCI still remains controversial.

**Methods:**

In the present study, we preliminary investigated the serum levels of interleukin (IL)-4, IL-10, major histocompatibility complex (MHC)-I, and inducible nitric oxide synthase (iNOS) and compared the serum IL-4 and IL-10 expression in rats of high Basso-Beattie-Bresnahan (BBB) scores with these of low BBB scores. Besides, the infiltration of macrophage and the axonal regeneration of the injured spinal cord were observed from day 10 to day 30.

**Results:**

We found that higher serum levels of IL-4 and IL-10 can reflect the restorability degree of SCI and could be potential biomarkers for the prognosis of SCI. The infiltration of the M2 subtype of macrophage and the axons regrowth might contribute to a better prognosis.

**Conclusions:**

The current study demonstrates that the serum levels of IL-4 and IL-10 are preliminarily adopted as serologic markers to forecast SCI, and high serum levels of IL-4 and IL-10 may indicate a better prognosis. Moreover, the way to promote macrophage polarization from M1 to M2 may contribute to better axonal regeneration.

## Background

Spinal cord injury (SCI) caused by trauma often leads to the disruption of axonal tracts, being the third most common cause of acquired disability worldwide [1, 2]. After the initial traumatic insult, the cascade reaction of blood supply reduction, oxidation, and inflammation following SCI leads to neurological disorders, including the destruction of the blood-spinal cord barrier, neuroinflammation, and oxidative stress [3, 4]. Inflammation in the early stage of SCI promotes the recovery of the injured spinal cord, and prematurely, glial scar or inflammation inhibition might lead to more serious damage to the injured spinal cord [5]. It is believed that different phenotypes of inflammatory cells play critical roles in neuroprotection. Neuroinflammation occurs almost immediately after spinal cord injury, including the activation of immune cells and cytokines [6]. Studies have demonstrated that mast cells, bone marrow-derived macrophages, dendritic cells, type 2 innate lymphocytes (ILC2s), and T cells might influence the outcome of spinal cord injury [7–11]. These cells release a large number of inflammatory factors (IL-1, IL-6, IL-8, IL-12, IFN-α, IFN-γ), chemokines (CXCL-1, CXCL-2), proteolytic enzymes, and complement proteins when damage occurs [12, 13]. Among these cytokines, the pro-inflammatory mediators, reactive oxygen species, and nitric oxide (NO) often contribute to inflammation and aggravate the traumatic injury [14]. On the contrary, the interleukin (IL)-4, IL-10, and IL-13 are pro-regenerative cytokines which can contribute to tissue repair, wound healing, and promote axons regeneration [15–17].

At present, spinal cord injury in rats is the most commonly used model in animal experiments of SCI, and the Basso-Beattie-Bresnahan (BBB) score is the most commonly used behavioral index to evaluate the recovery of lower limb motor function after SCI, which is also the most widely used evaluation standard of lower limb motor function in the world [18, 19]. The BBB locomotor rating scale is a 21-point scale, which was originally used in SCI rat models; it has high sensitivity, good test-retest reliability, and strong validity [19, 20]. Inter-rater reliability tests show that examiners with different experiences in behavioral tests can consistently use the scale and get similar scores [19]. The BBB score included almost all the behavioral changes of rats during the recovery of lower limb motor function after SCI, which was highly consistent with the degree of SCI.

Despite therapeutic strategies and imaging diagnostic techniques have improved dramatically in the past decades, the prognostic evaluation of SCI still depends on computed tomography (CT), magnetic resonance imaging (MRI), and the physical examination of neurological functions [21, 22]. Few studies have examined proinflammatory cytokines and immune cells to evaluate the prognosis of patients with SCI. In the current study, we evaluated the prognostic value of the serum IL-4 and IL-10 expression and observed the inflammatory cells of the injured spinal cord in predicting the prognosis of neurological function in patients with SCI.

## Materials and methods

### Animal breeding

Thirty-two male Sprague-Dawley rats, aged 6~8 weeks, weighing 220–250 g, were housed in the Laboratory Animal Centre of Nanjing Medical University in accordance with the animal experimental guidelines set by and with the approval of the National Institute of Health and the Nanjing Medical University.

### SCI procedures

The rats were intraperitoneally injected with 1% pentobarbital sodium (50 mg/kg). The model of SCI was established as previously described [23]. Briefly, the spinous process and the vertebral lamina were removed to expose the spinal cord in a circular region at the T10 level. A graduated force of 70 g was placed over the exposed dura and left for 60 s to induce a compression injury. The rat models of SCI should meet the following characteristics to make sure all the models were in a similar base level, including congestive edema of T10 segment, twitch of the tail and hindlimb, and neurogenic bladder. Each rat was feed separately, and the bladder of rats was given artificial massage twice a day until a reflex bladder emptying was established. Sham-operated mice received laminectomy without compression injury.

### Basso-Beattie-Bresnahan score

Lower limb motor function was evaluated using the BBB score, as previously described by Basso et al. [24]. The rats were placed in an open field and two independent; blinded examiners observed these rats for 10 minutes individually. The rats were tested at days 5, 10, 15, 20, 25, and 30 post-SCI surgery. In brief, the BBB scores of hindlimb motor function of SCI rats ranged from 0 (complete paralysis) to 21 (unimpaired locomotion), in which 0–7 mainly evaluated the movement of the hindlimb joints, 8–13 mainly evaluated the gait and coordination of the hindlimbs, and 14–21 mainly evaluated the fine movements of the claws in the movement [19, 20].

### Study design

The motor function of the lower limbs in all rats was evaluated using the BBB score after SCI operation at days 5, 10, 15, 20, 25, and 30, but the three rats with the highest BBB score (BBB high group) and the three with the lowest BBB score (BBB low group) were executed at days 10, 20, and 30. Therefore, the BBB high group always had three rats with the highest BBB score, and the BBB low group always had three rats with the lowest BBB score in every BBB score testing. The spinal cord tissues of the executed rats were removed and used for immunofluorescence detection in the BBB high group and BBB low group at days 10, 20, and 30, respectively.

### Serum evaluation

Blood samples were taken from the eyeballs of all surviving rats for serum evaluation at days 5, 10, 15, 20, 25, and 30. Serum levels of interleukin (IL)-4, IL-10, major histocompatibility complex (MHC)-I, and inducible nitric oxide synthase (iNOS) were detected using enzyme-linked immunosorbent assay (ELISA) according to the manufacturer’s instructions. The final concentrations of IL-4, IL-10, MHC-I, and iNOS were interpolated from the determined standard curve of absorbance. After discovering that there may be some kind of pattern, we further compared serum IL-4 and IL-10 levels of the three rats with the highest BBB score (BBB high group) and the three with the lowest BBB score (BBB low group) at days 5, 10, 15, and 30, respectively.

### Immunofluorescence

Spinal cord tissues were deparaffinized and rehydrated in coronal sections (4 μm thickness); then, the sections were blocked with 5% bovine serum albumin at room temperature for 30 min and then washed with PBS three times for 10 min. The sections were then incubated with corresponding primary antibodies overnight at 4 °C, including F4/80, iNOS, arginase (Arg)1, and NF200 (Invitrogen, Carlsbad, CA, USA), and incubated with secondary antibody (1:500; Abcam, Shanghai, China) for 3 h. The slides were visualized using a fluorescence microscope (Olympus BX 51, Tokyo, Japan), and ImageJ (Wayne Rasband, National Institutes of Health, Bethesda, MD, USA) was used for quantitative analysis.

### Statistical analysis

All data were presented as mean ± standard error of mean (SEM) and were analyzed by two-way *ANOVA* repeated measurement using *SPSS Statistics 20.0* (SPSS Inc., Chicago, IL, USA) for statistical significance. A *P* value < 0.05 was considered statistically significant.

## Results

### The change of cytokines in serum is related to the prognosis

**Fig. 1:**
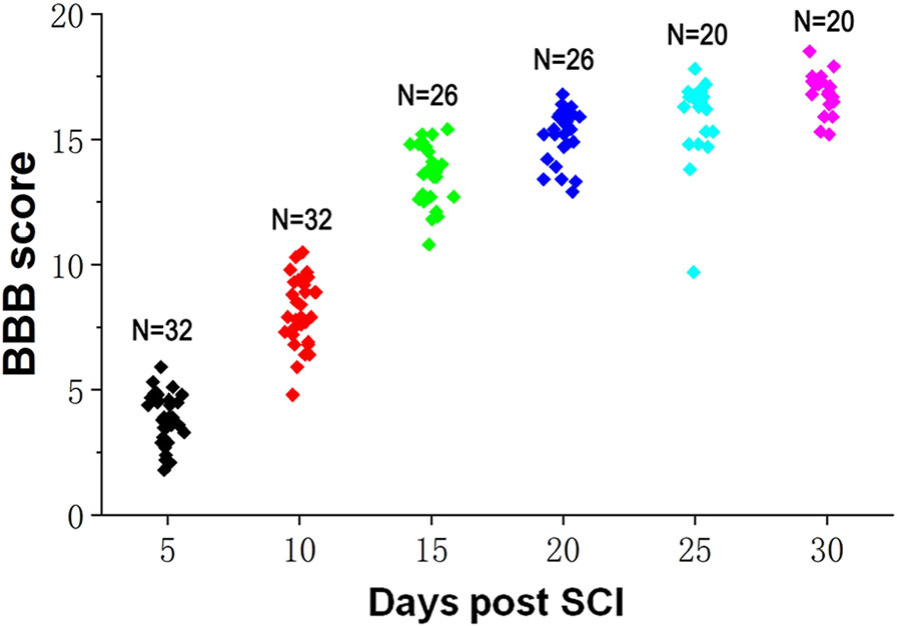
BBB scores of rats following SCI over time

**Fig. 2:**
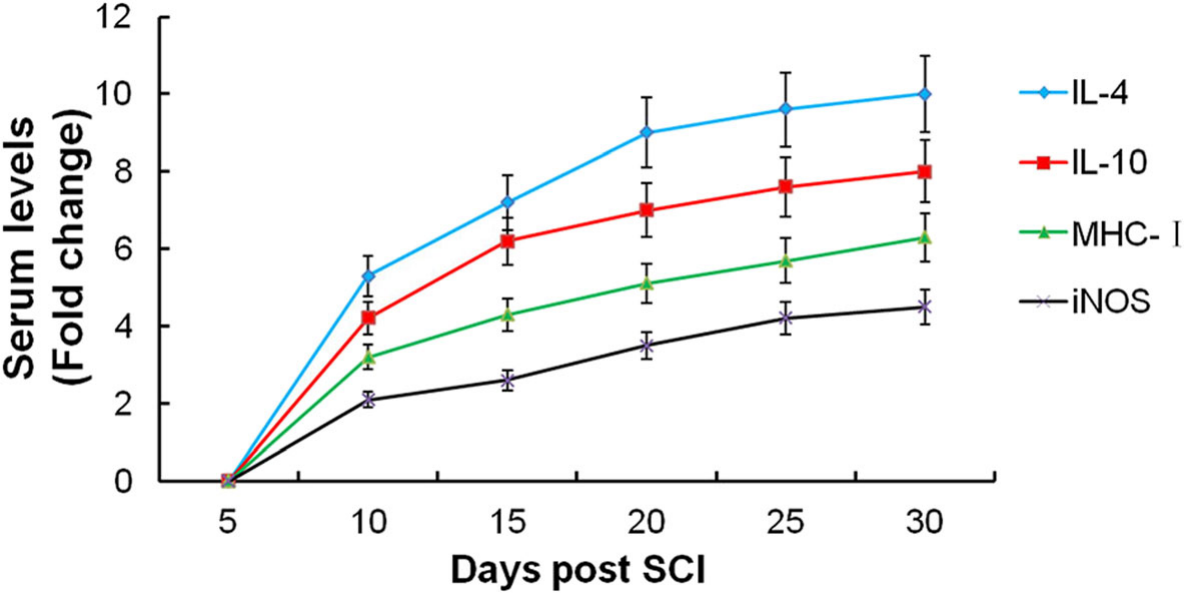
The serum levels of cytokines (IL-4, IL-10, MHC-I, and iNOS) were quantified by ELISA over time following SCI

**Fig. 3:**
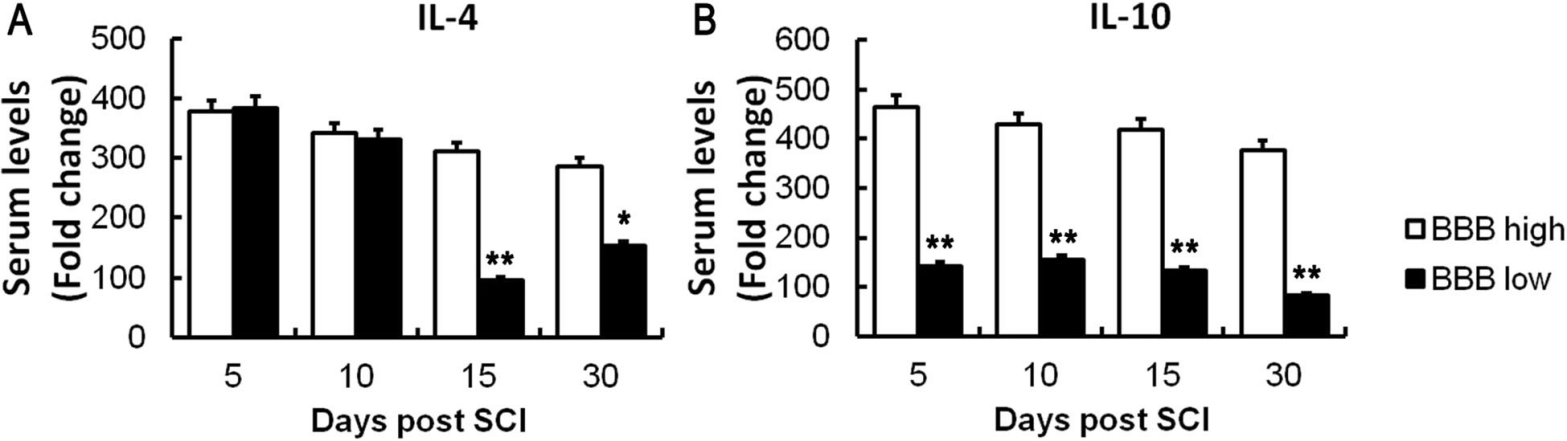
Comparison of serums IL-4 and IL-10 in the BBB high group and BBB low group. The serum levels of IL-4 (**a**) and IL-10 (**b**) were significantly higher in the BBB high group. *P < 0.05, **P < 0.01, as compared with the BBB high group

The SCI model was successfully established and all rats survived and were included in the statistical analysis. Our findings suggested that the lower limb motor function of rats gradually recovered following SCI, and the BBB score increased significantly during follow up (Fig. 1). Elisa revealed that the levels of serum IL-4, IL-10, MHC-I, and iNOS grew steadily from day 5 to day 30 after SCI, among which, the fold changes of IL-4 and IL-10 were the most obvious (Fig. 2). Therefore, we speculated that serum levels of IL-4 and IL-10 might change in rats with different prognoses. Ulteriorly, we compared the serum levels of IL-4 and IL-10 in the BBB high group and BBB low group, and we found that the serum levels of IL-4 and IL-10 in the BBB high group were significantly higher than those in the BBB low group (Fig. 3). These results suggested that IL-4 and IL-10 might be effective cytokines for predicting the prognosis of SCI, which needs further experiments to confirm.

### The polarization of macrophage predicts the prognosis of SCI

**Fig. 4:**
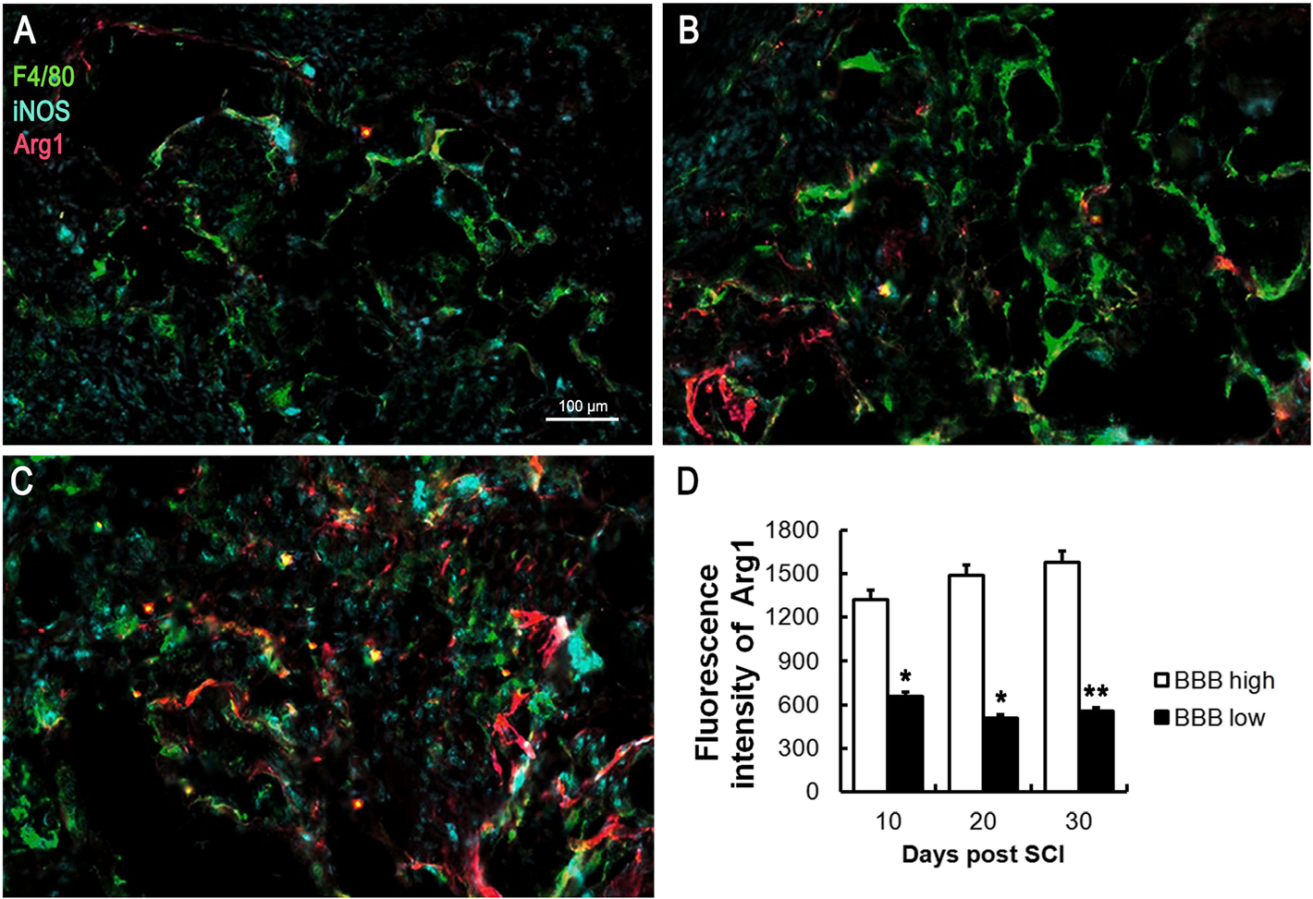
Polarized macrophages were observed by immunofluorescence following SCI. The number of macrophages in the damaged spinal cord rose dramatically at day 10 (**a**), day 20 (**b**), and day 30 (**c**) after SCI. The M2 subtype of macrophage in the BBB high group is significantly higher than that in the BBB low group from day 10 to day 30 following SCI (**d**). *P < 0.05, **P < 0.01, as compared with the BBB high group

Macrophage plays a critical role in the inflammatory response following SCI; therefore, the macrophage polarization was analyzed. F4/80 is a definite marker of macrophages. iNOS indicates that macrophages polarize to M1, and Arg1 indicates that macrophages polarize to M2. Immunofluorescence showed the F4/80 expression increased significantly from day 10 to day 30, indicating that the infiltration of macrophage in the injured spinal cord increased along with the time after SCI (Fig. 4a–c). Besides, the fluorescence intensity of Arg1 was upregulated, and iNOS was downregulated markedly from day 10 to day 30, indicating that macrophages polarize from M1 to M2 along with the time following SCI. Additionally, the fluorescence intensity of Arg1 in the BBB high group was significantly higher than that in the BBB low group (Fig. 4d). These results indicate that the upregulation of the M2 subtype of macrophage may predict a better prognosis following SCI.

### The axonal regrowth was activated after SCI

**Fig. 5:**
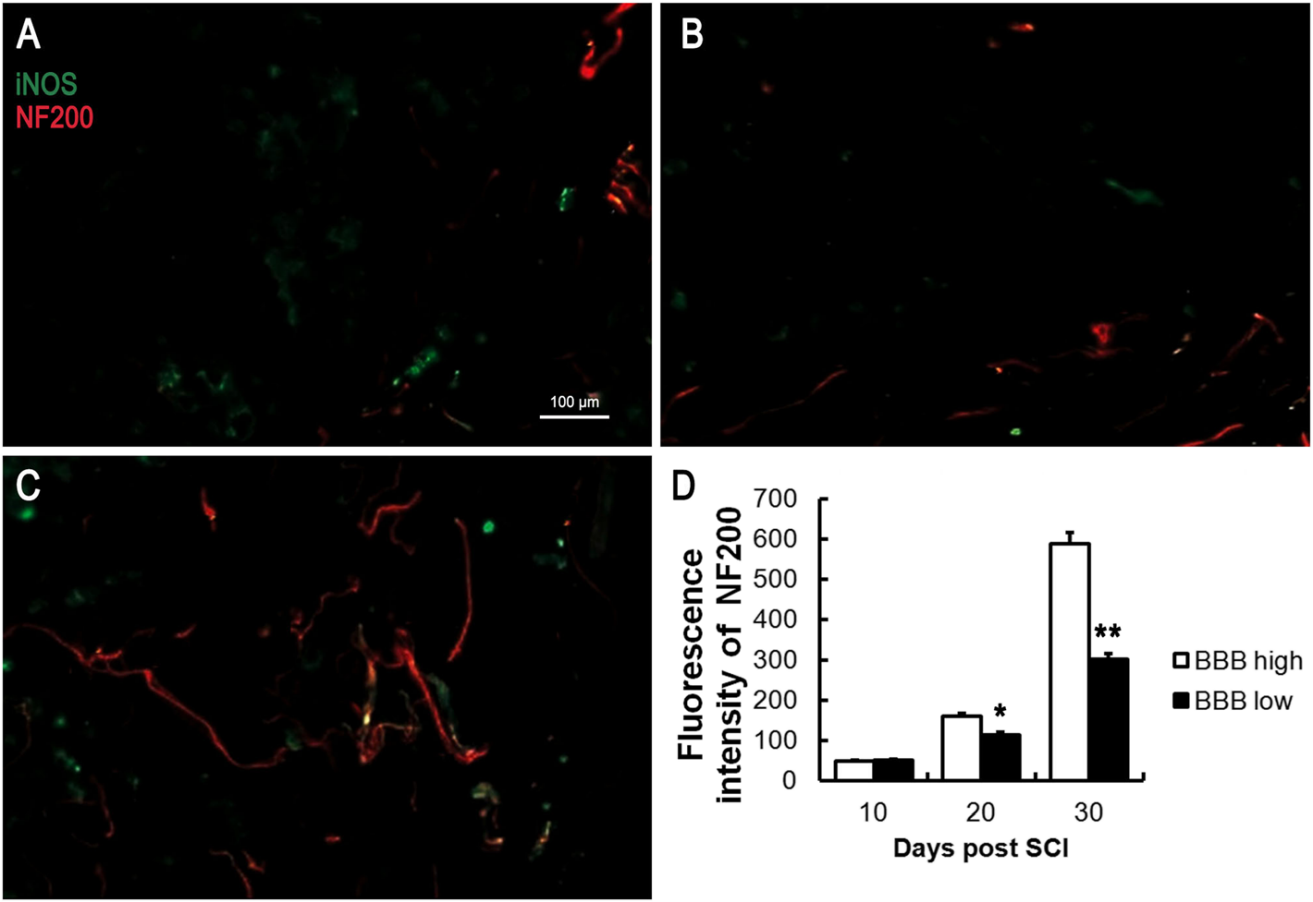
Axons were observed by immunofluorescence following SCI. The number of axons in the injured spinal cord increased at day 10 (**a**), day 20 (**b**), and days 30 (**c**) following SCI. The reborn axons in the BBB high group were markedly increased from day 20 to day 30 following SCI (**d**). *P < 0.05, **P < 0.01, as compared with the BBB high group

Axon regeneration promotes lower limb motor function after spinal cord injury in rats. To observe axonal regeneration in spinal cord tissue, NF200 was determined by immunofluorescence. NF200 is a specific marker of axonal regeneration. Our findings suggested that the fluorescence intensity of NF200 increased significantly from day 10 to day 30, indicating that axon regeneration increased along with the time following SCI (Fig. 5a–c). Besides, the fluorescence intensity of NF200 was upregulated markedly from day 10 to day 30, indicating that fluorescence intensity of NF200 in the BBB high group was significantly higher than that in the BBB low group (Fig. 5d).

## Discussion

Auxiliary examinations such as imaging and electrophysiological examinations are frequently used to evaluate SCI severity but fail to predict the prognosis [25, 26]. Therefore, a quantitative index must be established to allow the objective evaluation of SCI severity and prognosis. An inflammatory response at the injured spinal cord leads to compress within the vertebral column and results in secondary damage [27–29]. The pro-inflammatory cytokines such as MHC-I and nitric oxide (NO) all contribute to inflammation and tissue damage [30]. By contrast, the pro-regenerative cytokines including IL-4 and IL-10 contribute to wound healing and tissue repair and enhance regrowth of axons [31]. However, few studies have explored their roles in assessing SCI severity in rats with spine trauma.

The BBB score is an effective evaluation method for the recovery of lower limb motor function after spinal cord injury. A higher BBB score indicates better prognosis. In the present study, a compression injury of the spinal cord was successfully established in rats, and we found that the BBB score increased over time, indicating that the lower limb motor function of rats gradually recovered following SCI. Interestingly, we found that the serum levels of IL-4, IL-10, MHC-I, and iNOS grew steadily from day 5 to day 30 following SCI, especially the fold changes of IL-4 and IL-10 exhibit the most obvious. To explore the difference of cytokines between a high BBB score and low BBB score, 3 rats with the highest BBB score and the 3 with the lowest BBB score were selected at day 10, day 20, and day 30. We found that the serum levels of IL-4 and IL-10 in rats with high BBB scores were significantly higher than those in rats with low BBB scores, indicating that serums IL-4 and IL-10 might be effective cytokines for predicting the prognosis of SCI. To observe the inflammation and axon regeneration of the injured spinal cord at the pathological level, the protein expression of F4/80, iNOS, Arg1, and NF200 were assessed by immunofluorescence. F4/80 is a definite marker of macrophages [32]. iNOS indicates that macrophages polarize to M1, and Arg1 indicates that macrophages polarize to M2 [33]. NF200 is a skeletal structure of neural cells and axons, a specific marker of axonal regeneration, which can reflect the degree of axonal regeneration in the spinal cord of rats after SCI [34]. Our findings suggested that the expression of F4/80 was increased following SCI, suggesting that macrophages were infiltrated in the injured spinal cord. As time went on and with the increasing of BBB score, Arg1 expression increased, while iNOS decreased, suggesting that macrophages polarized from M1 to M2. Besides, NF200 expression increased significantly over, indicating that axon regeneration increased following SCI. Moreover, the fluorescence intensity of NF200 was upregulated effectively over time, indicating that axon regeneration in rats with high BBB scores was significantly higher than that in rats with low BBB scores.

Anti-inflammatory cytokines play a critical role in axon regeneration following SCI. In the neuroinflammatory microenvironment, the pro-inflammatory cytokines are cytotoxic to neurons, which lead to extend short neurite sprouts after SCI [15, 35]. IL-10 has been demonstrated to upregulate anti-apoptotic factors such as B cell lymphoma 2 (Bcl-2) and provide a direct trophic influence on neurons to improve the neurotoxic microenvironment [36, 37]. IL-4 in the SCI phase reduces the release of pro-inflammatory factors at the impaired spinal cord [38, 39]. Moreover, IL-10 has shown to induce the expression of IL-4R, and stimulate M2 subtype of macrophages to produce both IL-10 and IL-4, which may create a feed-forward process at the injured spinal cord site [17, 40]. As previous studies have demonstrated a change from M1 to M2 is an essential part of the repair process, and polarization of macrophages from M1 to M2 may contribute to a better axon regeneration and prognosis [41–43].

## Conclusions

Collectively, the current study demonstrates that the serum levels of IL-4 and IL-10 are preliminarily adopted as serologic markers to forecast SCI, and high serum levels of IL-4 and IL-10 may indicate a better prognosis. Moreover, the way to promote macrophage polarization from M1 to M2 may contribute to better axonal regeneration. There might be some correlations between serum IL-4, IL-10, and macrophage polarization in regulating axon regeneration, and the potential mechanisms need further exploration.

## Data Availability

Research data can be obtained from the corresponding authors upon reasonable request.

## Abbreviations

Arg: Arginase
BBB: Basso-Beattie-Bresnahan
Bcl-2: B cell lymphoma 2
CT: Computed tomography
ELISA: Enzyme-linked immunosorbent assay
IL-10: Interleukin-10
IL-4: Interleukin-4
ILC2s: Type 2 innate lymphocytes
iNOS: Inducible nitric oxide synthase
MHC-I: Major histocompatibility complex-I
MRI: Magnetic resonance imaging
NO: Nitric oxide
SCI: Spinal cord injury
SEM: Standard error of mean
